# Supplementary material for: Controlling the pandemic during the SARS-CoV-2 vaccination rollout
Source: Nat Commun. 2021 Jun 16;12:3674. doi: 10.1038/s41467-021-23938-8 (PMC8209021; doi:10.1038/s41467-021-23938-8)
Supplement: Supplementary file 1 — Supplementary Information [file 41467_2021_23938_MOESM1_ESM.pdf]

Supplementary Information

Controlling the pandemic during the SARS-CoV-2 vaccination rollout

João Viana, MSc[1,2], Christiaan H. van Dorp, PhD[3], Ana Nunes, PhD[2,4], Manuel C. Gomes, PhD[2], Michiel van Boven, PhD[1], Mirjam E. Kretzschmar, PhD[1], Marc Veldhoen, PhD[5], and Ganna Rozhnova, PhD[*1,4]

[1]Julius Center for Health Sciences and Primary Care, University Medical Center Utrecht, Utrecht University, Utrecht, The Netherlands

[2]Faculdade de Ciências, Universidade de Lisboa, Lisbon, Portugal

[3]Theoretical Biology and Biophysics (T-6), Los Alamos National Laboratory, Los Alamos, New Mexico, USA

[4]BioISI—Biosystems & Integrative Sciences Institute, Faculdade de Ciências, Universidade de Lisboa, Lisbon, Portugal

[5]Instituto de Medicina Molecular João Lobo Antunes, Faculdade de Medicina, Universidade de Lisboa, Lisbon, Portugal

May 20, 2021

*Corresponding author:

Dr. Ganna Rozhnova

Julius Center for Health Sciences and Primary Care

University Medical Center Utrecht

P.O. Box 85500 Utrecht

The Netherlands

Email: g.rozhnova@umcutrecht.nl

Phone: +31 631117965

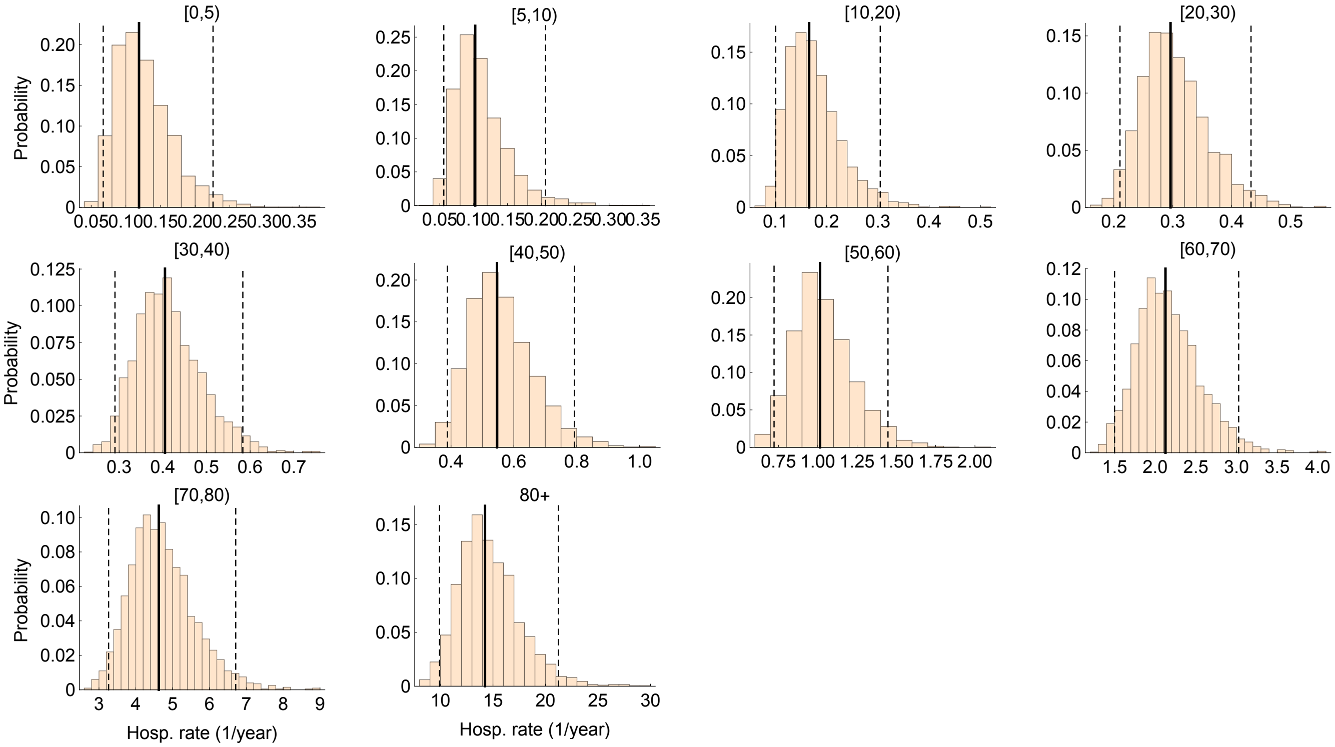

Supplementary Figure 1. Estimated hospitalization rates. The histograms of age-specific hospitalization rates estimated by the model in the Bayesian framework. Supplementary Table 4 indicates prior distributions of these parameters. The solid and the dashed lines are, respectively, the medians and the 95% credible intervals based on 2,000 parameter samples from the posterior distribution.

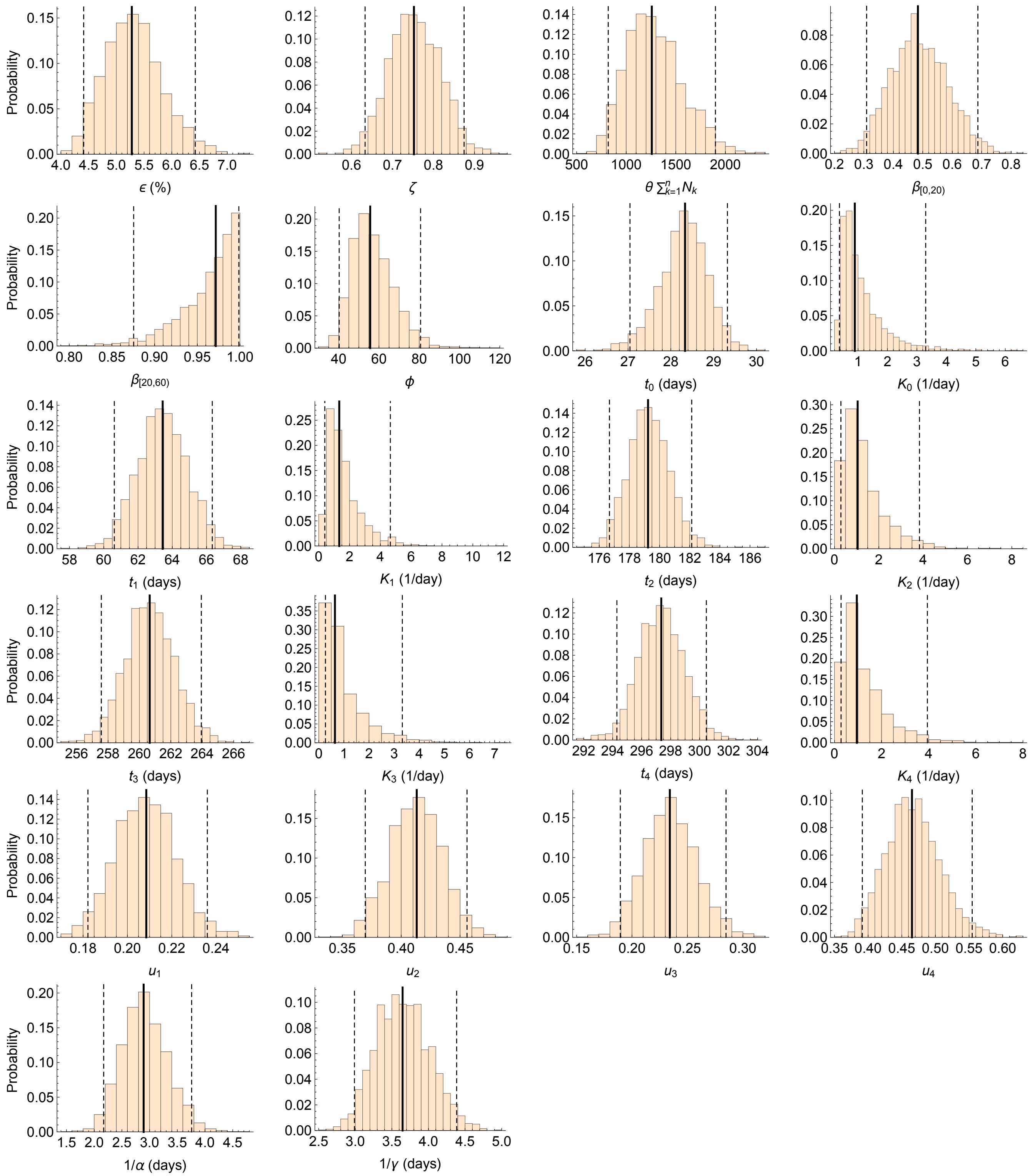

Supplementary Figure 2. Estimated model parameters. The histograms of parameters estimated by the model in the Bayesian framework. Supplementary Table 4 indicates prior distributions of these parameters. The solid and the dashed lines are, respectively, the medians and the 95% credible intervals based on 2,000 parameter samples from the posterior distribution. The time $t = 0$ corresponds to 26 February 2020. The susceptibility of 60+ age group was used as the reference, i.e. $\beta_{60+} \equiv 1$. The description of the parameters is given in Supplementary Table 1.

Supplementary Table 1. Summary of the model parameters.

Description (unit)	Notation	Reference
**Constant parameters**		
Latent period (days)	$1/\alpha$	Estimated
Infectious period (days)	$1/\gamma$	Estimated
Over-dispersion parameter for the NegBinom distribution for hospitalizations	ϕ	Estimated
Initial fraction of infected persons	θ	Estimated
Probability of transmission per contact	$\epsilon[1+0.5/(1+e^{-K_0(t-t_{\text{data}})})]^{\text{b}}$	ϵ and K_0 estimated, t_{data} is 15 Jan 2021
**Age-specific parameters**[a]		
Force of infection for unvaccinated and vaccinated persons (1/day)	$\lambda_k(t)$, $\lambda_k^V(t)$	Eqs. (3) and (4)
Contact rate for unvaccinated persons (1/day)	$c_{kl}(t)$	Estimated, see Suppl. Table 5
Contact rate for vaccinated persons (1/day)	$c_{kl}^V(t)$	Assumed
Hospitalization rate (1/day)	ν_k	Estimated
Susceptibility of age group k relative to age group 60+, $\beta_{60+} \equiv 1$	β_k	Estimated
Population size of age group k	N_k	[1]
**Vaccination parameters**[a]		
Vaccination rate (1/day)	r_k	Calculated from Table 1 and Figure 4 **a**
Vaccine efficacy in reducing susceptibility	VE_S	[2–9]
Vaccine efficacy in reducing infectivity	VE_I	[2–9]
Vaccine efficacy in reducing hospitalization rate	VE_H	[2–9]
**Vaccine efficacies sets**		
Optimistic set (Figures 6, 7, Suppl. Figures 5, 6, 10)		94%, 0%, 67%
Pessimistic set (Suppl. Figures 7, 8, Suppl. Table 2)		55%, 0%, 0%
Sensitivity to VE_I (Suppl. Figure 11, Suppl. Table 6)	(VE_S, VE_I, VE_H)	94%, 0%, 67%
		94%, 50%, 67%
		94%, 100%, 100%
		55%, 0%, 0%
		55%, 50%, 0%
		55%, 100%, 100%

Notes: [a]Indices k and l denote the age groups $k, l = 1, \ldots, n$, where $n = 10$ is the number of age groups. [b]The rapid spread of B.1.1.7 variant, that is estimated to be about 50% more transmissible based on the data from England [10–12], fueled the third wave of hospitalizations in Portugal. The increasing dominance of this variant was modelled empirically as a gradual increase in the probably of transmission per contact by 50% as follows $\epsilon[1+0.5/(1+e^{-K_0(t-t_{\text{data}})})]$, where ϵ and K_0 were estimated based on the data until 15 January 2021 (Supplementary Figure 2) and t_{data} is the last date in the hospital admission data (15 January 2021).

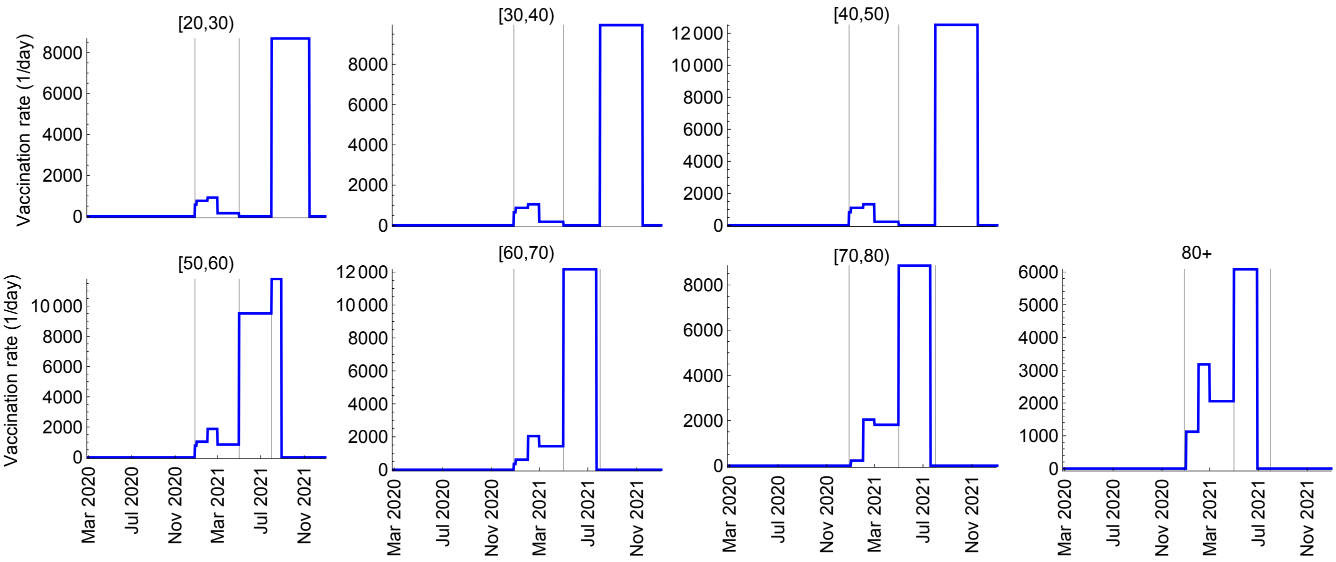

Supplementary Figure 3. Age-specific vaccination rates. Vaccination rate (number of persons vaccinated per day) per age group calculated using the national vaccination plan (Table 1) and age distribution of various vaccination categories (Figure 4 **a**). The vertical lines indicate the starting dates of different phases of vaccination (Table 1). According to the current guidelines persons under 18 years old are not eligible for vaccination. In the model, we assumed that the age group of 0 to 20 years old is not vaccinated.

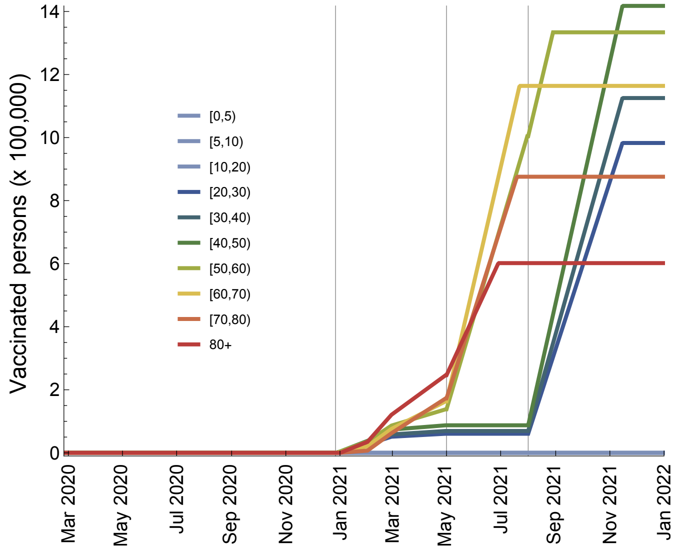

Supplementary Figure 4. Number of vaccinated persons per age group during the vaccination rollout. These numbers were calculated using the national vaccination plan (Table 1) and age distribution of various vaccination categories (Figure 4 **a**). The vertical lines indicate the starting dates for vaccination of different phases of vaccination (Table 1). According to the current guidelines persons under 18 years old are not eligible for vaccination. In the model, we assumed that the age group of 0 to 20 years old is not vaccinated.

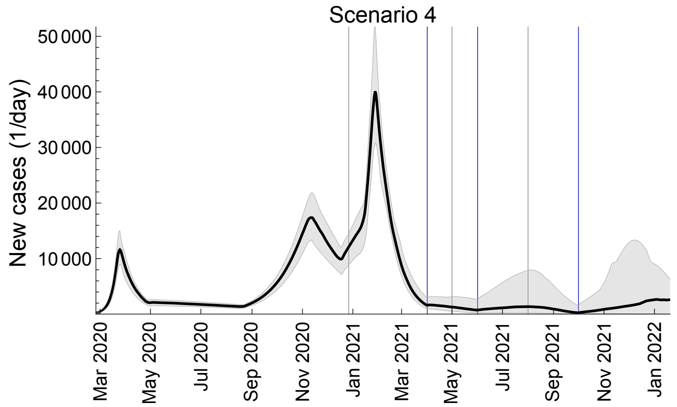

Supplementary Figure 5. Infectious cases dynamics for Figure 7. New daily cases of SARS-CoV-2 for Scenario 4 presented in Figure 7 in the main text. The black line is the median trajectory estimated from the model. The gray shaded region corresponds to 95% credible intervals. The blue vertical lines indicate the mid-points of relaxation steps (1 April, 1 June, 1 October). The gray vertical lines indicate the starting dates for different vaccination phases (Table 1).

Supplementary Table 2. Cumulative median hospitalizations and cases from 1 April 2021 till 24 June 2022.

Scenario 4*	Hospitalizations	Cases in vaccinated	Cases in unvaccinated
1. Optimistic vaccine efficacies	4,088	40,892	708,353
2. Optimistic vaccine efficacies & pre-pandemic contacts rates for vaccinated population	4,301	44,883	724,926
3. Pessimistic vaccine efficacies	30,028	1,200,810	1,390,640
4. Pessimistic vaccine efficacies & pre-pandemic contacts rates for vaccinated population	31,344	1,085,410	1,497,820

Notes: *The plots are shown in Supplementary Figures 7 and 8.

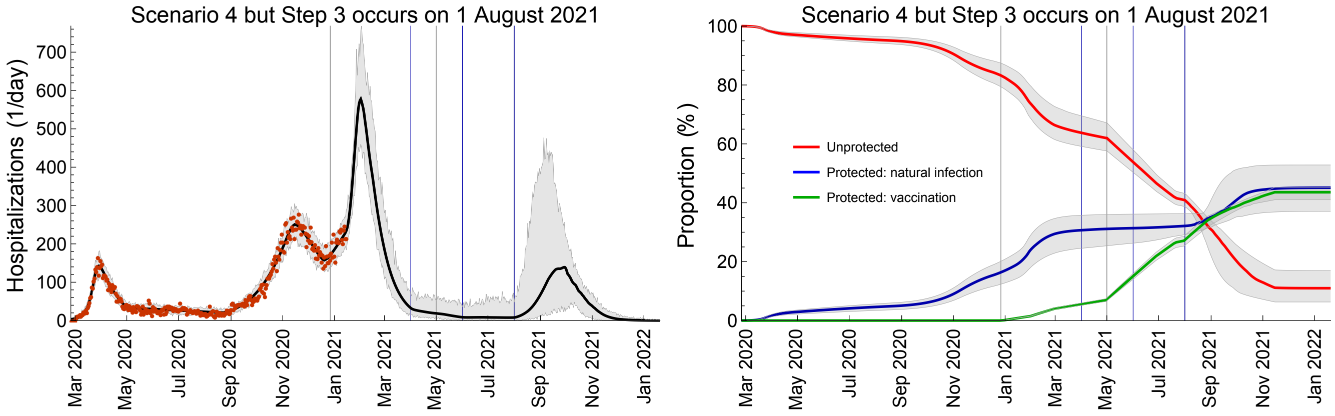

Supplementary Figure 6. Impact of timings of different relaxation steps. Total daily hospital admissions with COVID-19 and proportion of protected population for Scenario 4 (Figure 7 in the main text) with Step 3 occurring on 1 August instead of 1 October 2021. The hospitalization data are shown as red dots. The solid lines are the median trajectories estimated from the model. The gray shaded regions correspond to 95% credible intervals. The blue vertical lines indicate the mid-points of relaxation steps (1 April, 1 June, 1 August). The gray vertical lines indicate the starting dates for different vaccination phases (Table 1).

Supplementary Table 3. ICPC-2 codes for morbidities specified in the Portuguese vaccination plan.

Morbidities	ICPC-2 code
Cardiac insufficiency	K75, K77
Coronary heart disease	K74, K76
Renal insufficiency	U99 and GFR $<$ 60 ml/min
COPD	R95 or another chronic respiratory disease requiring ventilation
Diabetes	T89, T90
Neoplasm	A79, B72-74, D74-76, F74, H75, K72, L71, N74, R84-85, S77, T71, T73, U75-77, X75-77, Y77-78
Hepatic insufficiency	D97
Obesity	T82
High blood pressure	K86, K87

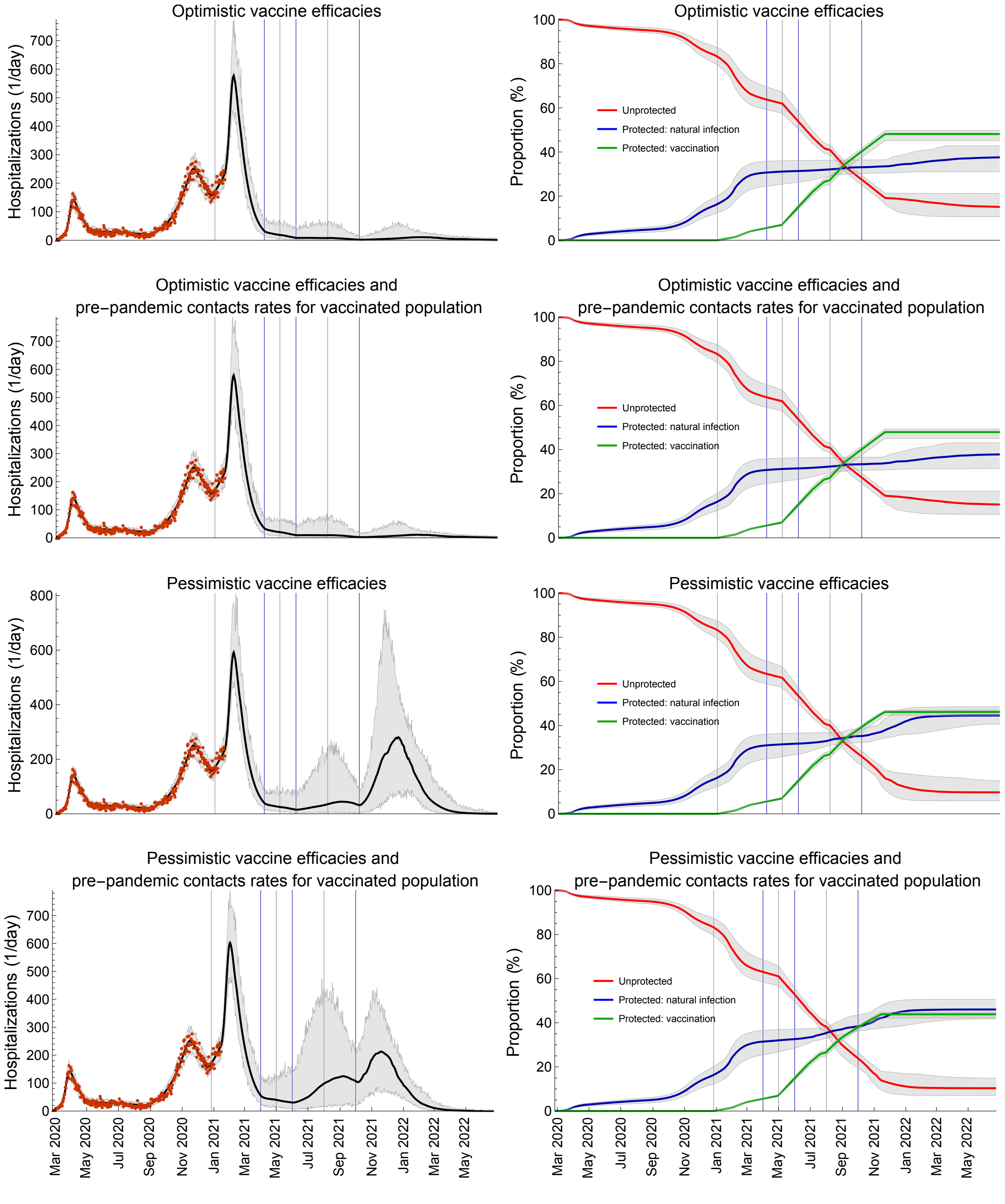

Supplementary Figure 7. Impact of vaccine efficacies and contact rates of vaccinated individuals. Scenario 4 but with an optimistic and a pessimistic set of vaccine efficacies (Supplementary Table 1). In addition to using two sets of vaccine efficacies, we allow for behavior compensation post-vaccination modelled as a return to pre-pandemic contact rates among vaccinated persons as compared to unvaccinated persons who may continue to have reduced contact rates due to control measures. For easier comparison of all scenarios, the top row panels (optimistic vaccine efficacies) are the same as Figures 7 **a** and **d** in the main text but plotted for a longer period of time (until 24 June 2022). The infectious cases dynamics is shown in Supplementary Figure 8 and the cumulative median number of hospitalizations is summarized in Supplementary Table 2. The hospitalization data are shown as red dots. The solid lines are the median trajectories estimated from the model. The gray shaded regions correspond to 95% credible intervals. The blue vertical lines indicate the mid-points of relaxation steps (1 April, 1 June, 1 October 2021). The gray vertical lines indicate the starting dates for different vaccination phases (Table 1).

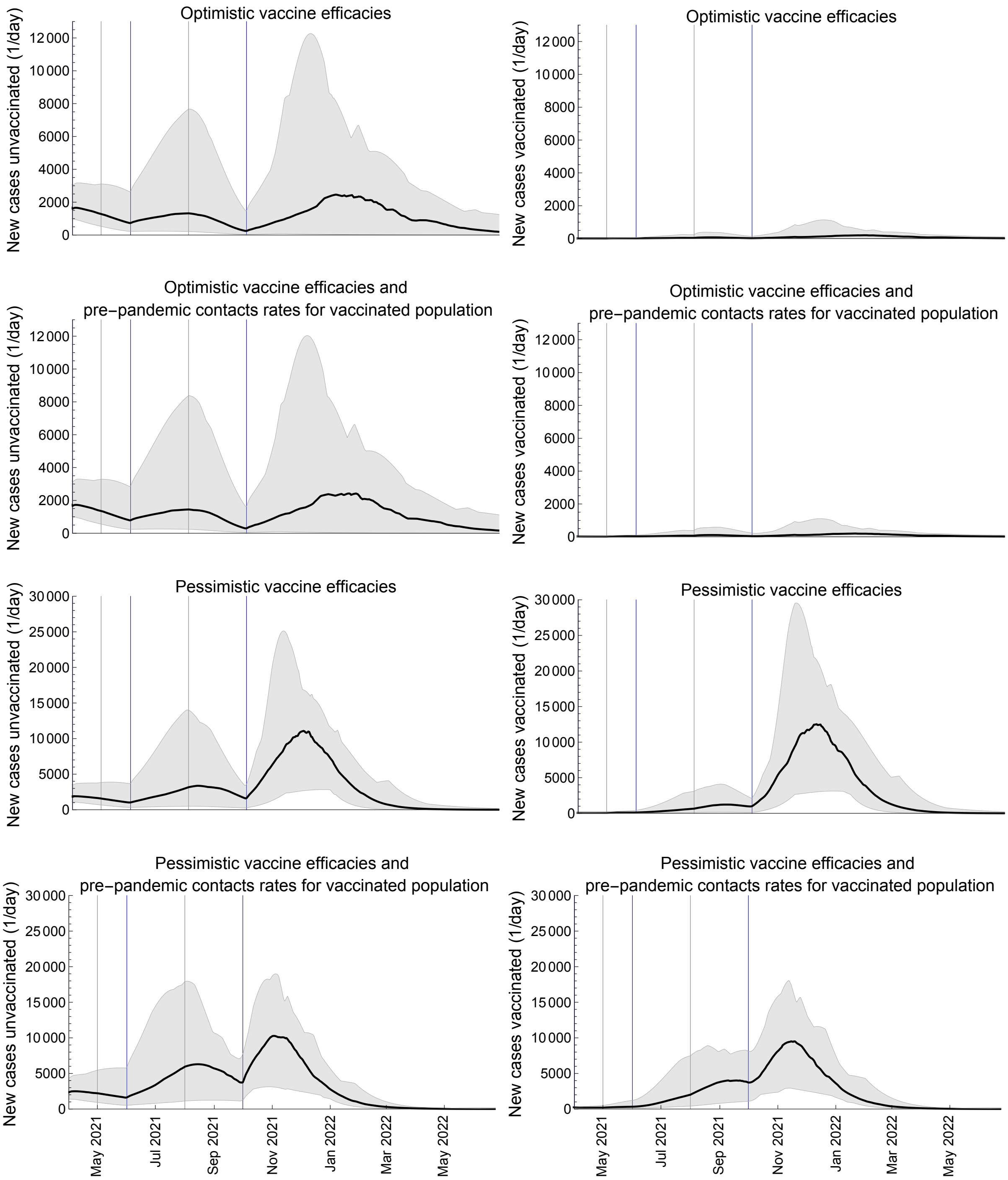

Supplementary Figure 8. Infectious cases dynamics for Supplementary Figure 7. New daily cases of SARS-CoV-2 in vaccinated and unvaccinated populations for scenarios presented in Supplementary Figure 7. These scenarios correspond to an optimistic and a pessimistic set of vaccine efficacies (Supplementary Table 1) and the possibility of behavior compensation post-vaccination modelled as a return to pre-pandemic contact rates among vaccinated persons as compared to unvaccinated persons who may continue to have reduced contact rates due to control measures. The cumulative median number of cases is summarized in Supplementary Table 2. The black line is the median trajectory estimated from the model. The gray shaded region corresponds to 95% credible intervals. The blue vertical lines indicate the mid-points of relaxation steps (1 April - coincides with the x-axis, 1 June, 1 October). The gray vertical lines indicate the starting dates for different vaccination phases (Table 1).

Supplementary Table 4. Prior distribution of the statistical model.

Parameter	Prior	Description
ϵ	Uniform$(0,1)$	Flat prior
θ	Uniform$(10^{-7}, 5\cdot 10^{-4})$	Vague prior allowing for 1 to 5000 infected individuals on day $t=0$
ϕ	Lognormal$(5,2)$	Vague prior[a]
α	InvGamma$(32.25, 9.75)$	99% of the prior density of α^{-1} is between 2 and 5 days;<br>chosen to be shorter than the incubation period [13, 14]
γ	InvGamma$(80, 20)$	99% of the prior density of γ^{-1} is between 2.9 and 5.2 days;<br>chosen to match the observed serial interval [15]
ν_k	folded-$\mathcal{N}(0,5)$	Vague prior, where k denotes $[0,5)$, $[5,10)$, $[10,20)$, $[20,30)$, $[30,40)$, $[40,50)$, $[50,60)$, $[60,70)$, $[70,80)$, 80+
$\beta_{[0,20)}$	LogNormal$(\log(0.23), 0.5)$	Odds-ratio[b] 2.23 based on prior estimates [16]
$\beta_{[20,60)}$	LogNormal$(\log(0.64), 0.5)$	Odds-ratio[b] 0.64 based on prior estimates [16]
ζ	$\mathcal{N}(1, 0.1)$	*A priori*, ζ should be close to 1
u_i	Uniform$(0,1)$	Flat prior $(i = 1, \ldots, 4)$
K_i	Exp(1)	With $K_i = 1$, the uptake of control measures takes approximately 6 days $(i = 0, \ldots, 4)$
t_0	$\mathcal{N}(22, 7)$	First lockdown around 18 March 2020 (State of Emergency)
$t_1 - 2.94/K_1$	$\mathcal{N}(69, 7)$	Start of relaxation of lockdown around 4 May 2020 2020[c]
t_2	$\mathcal{N}(203, 7)$	Further relaxation on 15 September 2020 (school opening)
t_3	$\mathcal{N}(254, 7)$	Second lockdown 05 November 2020 (State of Emergency)
t_4	$\mathcal{N}(304, 7)$	Relaxation of second lockdown on 25 December 2020

Notes: [a]The scale parameters of the normal distributions are equal to the standard deviation. [b]The age class 60+ is taken as a reference for the relative susceptibility, i.e., $\beta_{60+} \equiv 1$. [c]The prior on the time of relaxation of the first lockdown is put on the time where the logistic function equals 5%. Notice that $\text{logit}(0.05) = -2.94$.

Supplementary Table 5. Parameters describing contact structure.

Description (unit)	Notation*	Reference
**Contact rates (1/day)**		
Baseline (pre-pandemic)	b_{kl}	[17]
After the first lockdown	ζa_{kl}	ζ estimated, a_{kl} inferred using [18]
After the first relaxation	$u_1 b_{kl} + (1-u_1)\zeta a_{kl}$	Estimated
After the second relaxation due to school opening	$u_2 b_{kl} + (1-u_2)\zeta a_{kl}$	Estimated
After the second lockdown	$u_3 b_{kl} + (1-u_3)\zeta a_{kl}$	Estimated
After the relaxation due to winter holidays	$u_4 b_{kl} + (1-u_4)\zeta a_{kl}$	Estimated
After the third lockdown	ζa_{kl}	Assumed
After first relaxation during the vaccination rollout (Scenario 1)	b_{kl}	Assumed
After first relaxation during the vaccination rollout (Scenario 2)	$u_2 b_{kl} + (1-u_2)\zeta a_{kl}$	Assumed
After first relaxation during the vaccination rollout (Scenario 3)	$u_1 b_{kl} + (1-u_1)\zeta a_{kl}$	Assumed
After first, second, third relaxation during the vaccination rollout (Scenario 4)	Matrices for Scenario 3, 2, 1	Assumed
**Mid-point time of the logistic function (days)**		
Introduction of the first lockdown	t_0	Estimated
Relaxation after the first lockdown	t_1	Estimated
Second relaxation due to school opening	t_2	Estimated
Introduction of the second lockdown	t_3	Estimated
Relaxation due to winter holidays	t_4	Estimated
Introduction of the third lockdown	t_5	28 January 2021, Assumed
First relaxation during the vaccination rollout	t_6	1 April 2021, Assumed
Second relaxation during the vaccination rollout	t_7	1 June 2021, Assumed
Third relaxation during the vaccination rollout	t_8	1 October 2021 (main analyses), 1 August (sensitivity analyses), Assumed
**Slope of the logistic function (1/day)**		
Introduction of the first lockdown	K_0	Estimated
Relaxation after the first lockdown	K_1	Estimated
Second relaxation due to school opening	K_2	Estimated
Introduction of the second lockdown	K_3	Estimated
Relaxation due to winter holidays	K_4	Estimated
Introduction of the third lockdown	K_0	Assumed
First relaxation during the vaccination rollout	K_1	Assumed
Second relaxation during the vaccination rollout	K_1	Assumed
Third relaxation during the vaccination rollout	K_1	Assumed
**Proportion of time a person behaves as before the pandemic**		
Relaxation after the first lockdown	u_1	Estimated
Second relaxation due to school opening	u_2	Estimated
Introduction of the second lockdown	u_3	Estimated
Relaxation due to winter holidays	u_4	Estimated

*Indices k and l denote the age groups $k, l = 1, \ldots, n$, where $n = 10$ is the number of age groups.

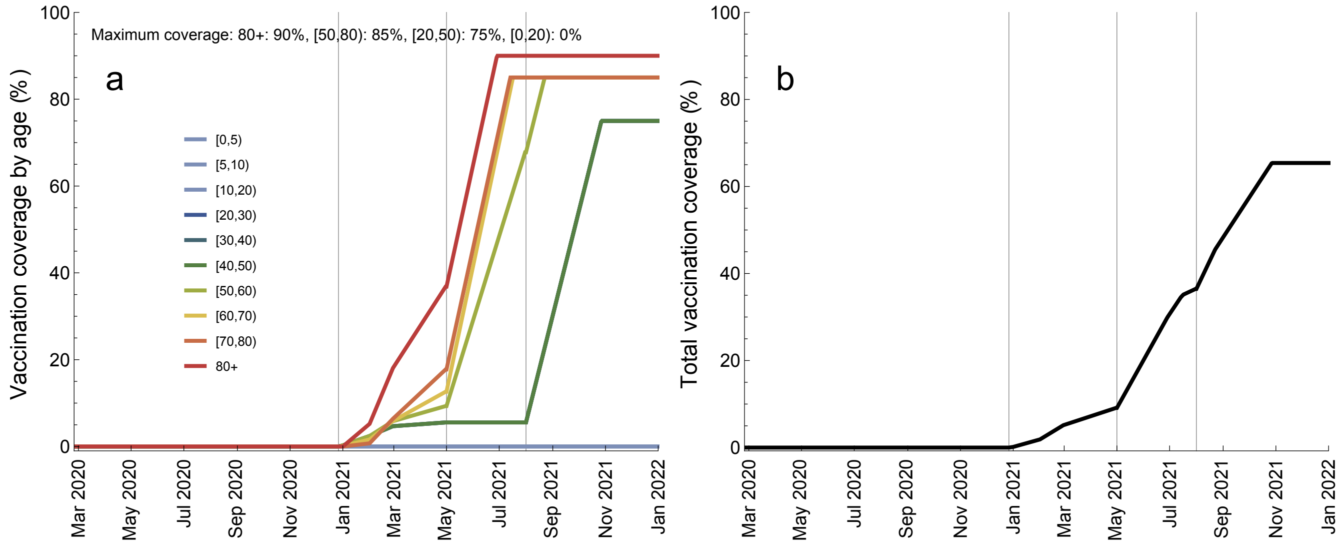

Supplementary Figure 9. Sensitivity analyses for the maximum vaccination coverage. a Age-specific coverage (percentage of vaccinated persons per age group). **b** Total vaccination coverage (percentage of vaccinated persons in the population). The gray vertical lines indicate the starting dates for different vaccination phases (Table 1). The maximum coverage for age group of [0,20), [20,50), [50,80), and 80+ is 0%, 75%, 85%, and 90%, respectively. The relaxation scenarios are shown in Supplementary Figure 10. For comparison, in the main text the maximum vaccination coverage for age group of [0,20) and 20+ is 0% and 90%, respectively (Figures 5, 6 and 7).

Supplementary Table 6. Cumulative median hospitalizations and cases from 1 April 2021 till 24 June 2022.

Sensitivity analyses to VE_I^*	Hospitalizations	Cases in vaccinated	Cases in unvaccinated
$VE_S = 94\%$, $VE_I = 0\%$, $VE_H = 67\%$	4,088	40,892	708,353
$VE_S = 94\%$, $VE_I = 50\%$, $VE_H = 67\%$	3,767	36,054	663,226
$VE_S = 94\%$, $VE_I = 100\%$, $VE_H = 100\%$	3,492	31,461	619,910
$VE_S = 55\%$, $VE_I = 0\%$, $VE_H = 0\%$	30,028	1,200,810	1,390,640
$VE_S = 55\%$, $VE_I = 50\%$, $VE_H = 0\%$	12,815	509,925	941,670
$VE_S = 55\%$, $VE_I = 100\%$, $VE_H = 100\%$	6,410	215,768	620,192

Notes: *The plots are shown in Supplementary Figure 11.

References

[1] Contemporary Portugal Database (Pordata); 2020. Available from: https://www.pordata.pt/.

[2] Polack FP, Thomas SJ, Kitchin N, Absalon J, Gurtman A, Lockhart S, et al. Safety and Efficacy of the BNT162b2 mRNA Covid-19 Vaccine. New England Journal of Medicine. 2020;383(27):2603–2615. doi:10.1056/NEJMoa2034577.

[3] Dagan N, Barda N, Kepten E, Miron O, Perchik S, Katz MA, et al. BNT162b2 mRNA Covid-19 Vaccine in a Nationwide Mass Vaccination Setting. New England Journal of Medicine. 0;0(0):null. doi:10.1056/NEJMoa2101765.

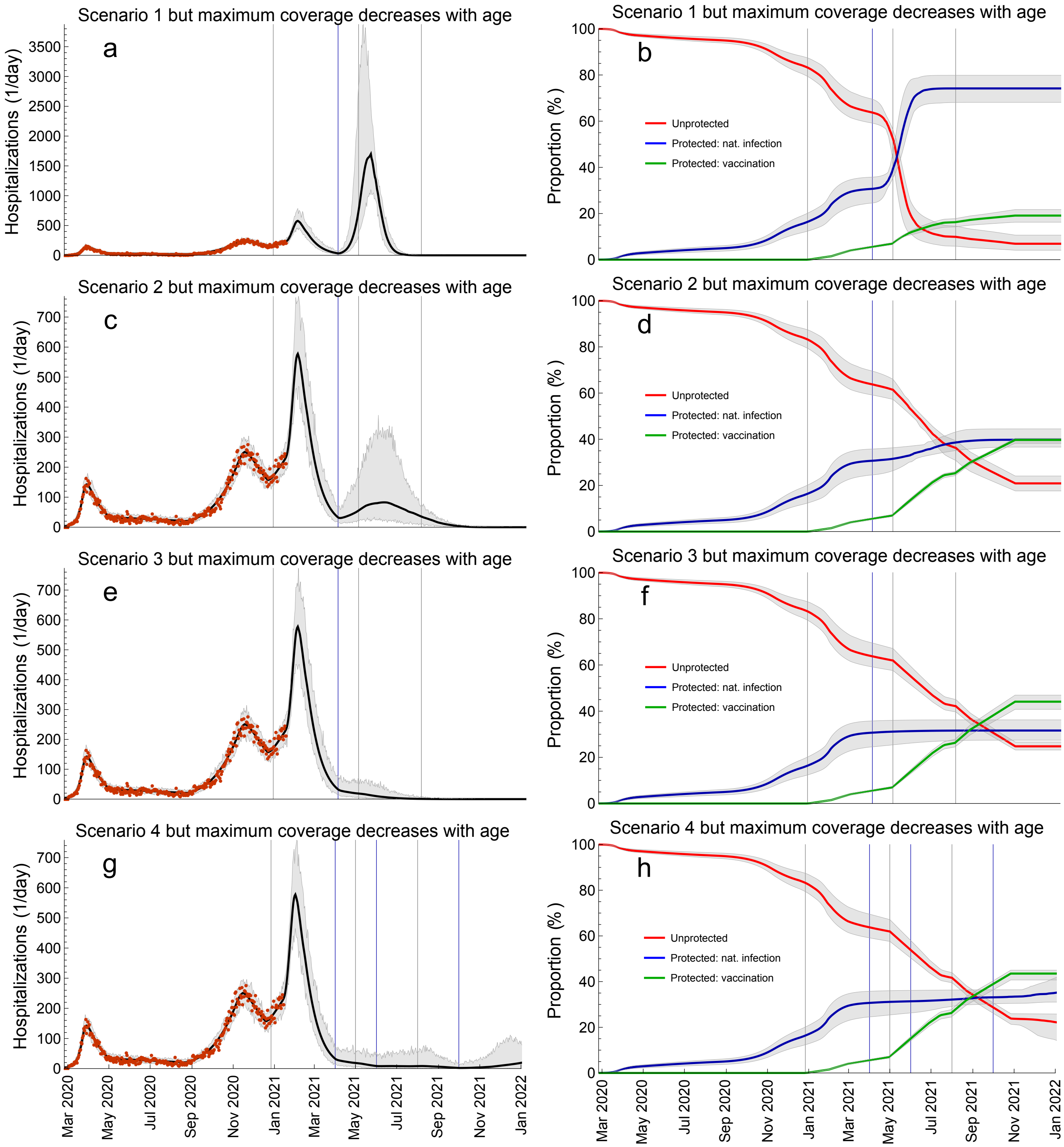

Supplementary Figure 10. Impact of the maximum vaccination coverage. Scenarios 1, 2, 3 and 4 (Figures 6 and 7 in the main text) but with the maximum vaccination coverage decreasing with age (see Supplementary Figure 9). The maximum coverage for age group of [0,20), [20,50), [50,80), and 80+ is 0%, 75%, 85%, and 90%, respectively. The hospitalization data are shown as red dots. The solid lines are the median trajectories estimated from the model. The gray shaded regions correspond to 95% credible intervals. The blue vertical lines indicate the mid-points of relaxation steps (1 April, 1 June, 1 October 2021). The gray vertical lines indicate the starting dates for different vaccination phases (Table 1). The cumulative median number of hospitalizations between 1 April 2021 and 1 January 2022 is practically the same for Scenarios 1, 2, 3, and 8% higher for Scenario 4 than in Figures 6 and 7.

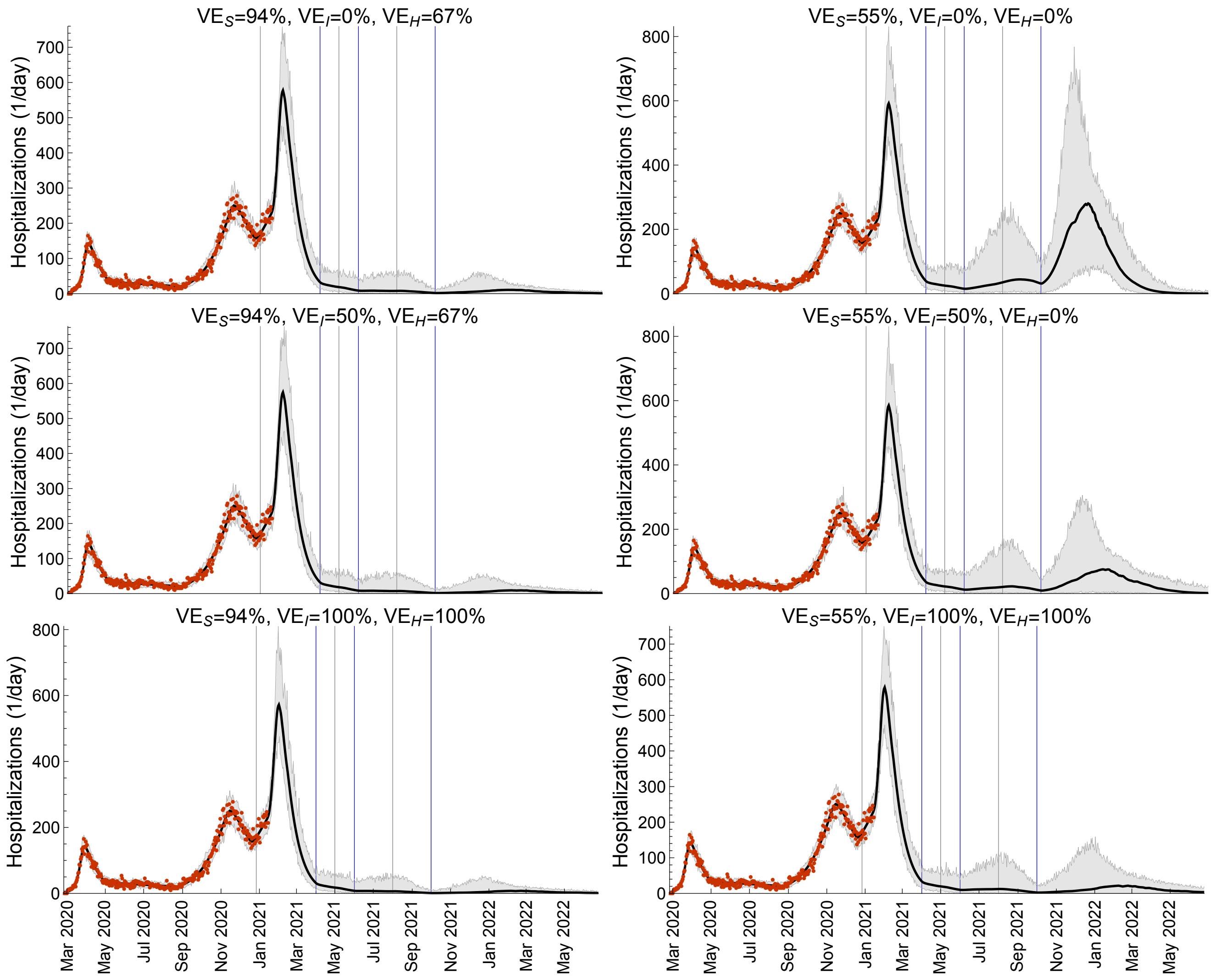

Supplementary Figure 11. Sensitivity analyses for $\mathbf{VE_I}$. We considered Scenario 4 (top row corresponding to optimistic and pessimistic assumptions about vaccine efficacies with $VE_I = 0\%$) and two additional values of VE_I (Supplementary Table 1): $VE_I = 50\%$ that corresponds to 50% infectivity of vaccinated persons relative to infectivity of unvaccinated persons and $VE_I = 100\%$ that is a best-case scenario implying that breakthrough cases in vaccinated individuals are not infectious at all. The hospitalization data are shown as red dots. The solid lines are the median trajectories estimated from the model. The gray shaded regions correspond to 95% credible intervals. The blue vertical lines indicate the mid-points of relaxation steps (1 April, 1 June, 1 October 2021). The gray vertical lines indicate the starting dates for different vaccination phases (Table 1). The cumulative median number of hospitalizations and cases in vaccinated and unvaccinated are summarized in Supplementary Table 6.

[4] Moustsen-Helms IR, Emborg HD, Nielsen J, Nielsen KF, Krause TG, Mølbak K, et al. Vaccine effectiveness after 1st and 2nd dose of the BNT162b2 mRNA Covid-19 Vaccine in long-term care facility residents and healthcare workers – a Danish cohort study. medRxiv. 2021;doi:10.1101/2021.03.08.21252200.

[5] Real-World Evidence Confirms High Effectiveness of Pfizer-BioNTech COVID-19 Vaccine and Profound Public Health Impact of Vaccination One Year After Pandemic Declared; 2021. Available from: https://www.businesswire.com/news/home/20210311005482/en/.

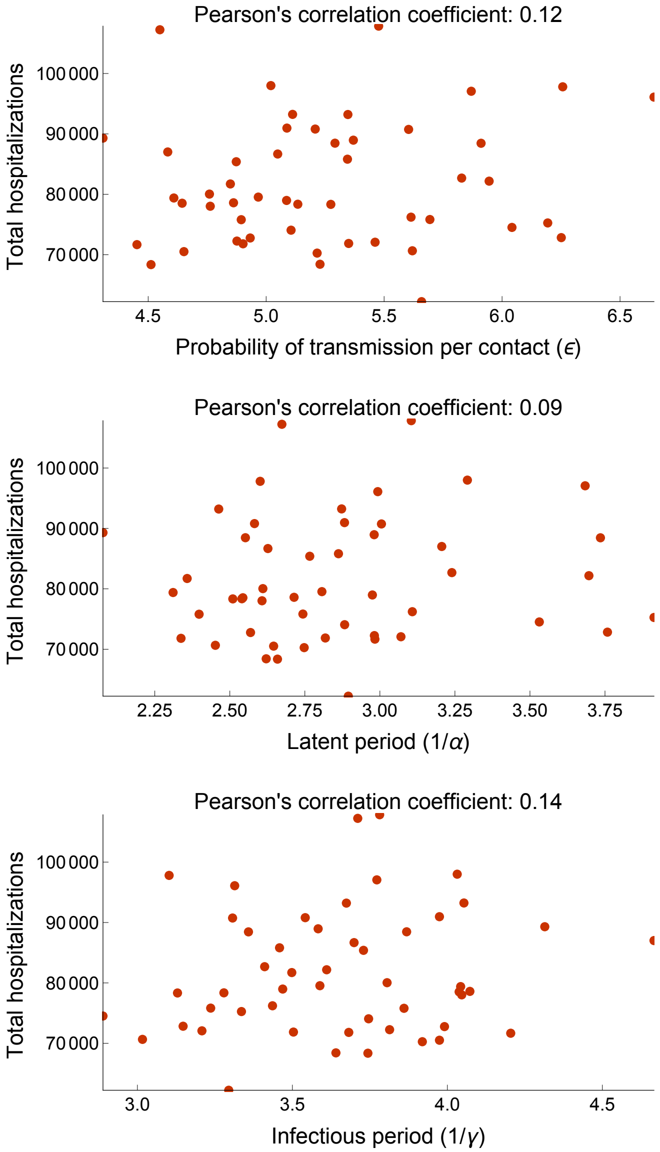

Supplementary Figure 12. Sensitivity of total hospitalizations to key epidemiological parameters. Scatter plots of the cumulative number of hospitalizations from 25 February 2020 till 24 June 2022 and the estimated probability of transmission per contact, the latent period and the infectious period for Scenario 4 and pessimistic assumptions about vaccine efficacies. Pearson correlation coefficients between the three parameters and the cumulative hospitalizations are in the range of 0.09 to 0.14.

[6] Hall, Victoria Jane and Foulkes, Sarah and Saei, Ayoub and Andrews, Nick and Oguti, Blanche and Charlett, Andre and Wellington, Edgar and Stowe, Julia and Gillson, Natalie and Atti, Ana and Islam, Jasmin and Karagiannis, Ioannis and Munro, Katie and Khawam, Jameel and Group, The SIREN Study and Chand, Meera A and Brown, Colin and Ramsay, Mary E and Bernal, Jamie Lopez and Hopkins, Susan. Effectiveness of BNT162b2 mRNA Vaccine Against Infection and COVID-19 Vaccine Coverage in Healthcare Workers in England, Multicentre Prospective Cohort Study (the SIREN Study); 2021. Available from: http://dx.doi.org/10.2139/ssrn.3790399.

[7] Chodick G, Tene L, Patalon T, Gazit S, Tov AB, Cohen D, et al. The effectiveness of the first dose of BNT162b2 vaccine in reducing SARS-CoV-2 infection 13-24 days after immunization: real-world evidence. medRxiv. 2021;doi:10.1101/2021.01.27.21250612.

[8] Scientific Advisory Group for Emergencies. Imperial College London: Unlocking roadmap scenarios for England, 18 February 2021; 2021. Available from: https://www.gov.uk/government/publications/imperial-college-london-unlocking-roadmap-scenarios-for-england-18-february-2021.

[9] Lipsitch M, Kahn R. Interpreting vaccine efficacy trial results for infection and transmission. medRxiv. 2021;doi:10.1101/2021.02.25.21252415.

[10] Davies NG, Abbott S, Barnard RC, Jarvis CI, Kucharski AJ, Munday JD, et al. Estimated transmissibility and impact of SARS-CoV-2 lineage B.1.1.7 in England. Science. 2021;doi:10.1126/science.abg3055.

[11] Volz E, Mishra S, Chand M, Barrett JC, Johnson R, Geidelberg L, et al. Transmission of SARS-CoV-2 Lineage B.1.1.7 in England: Insights from linking epidemiological and genetic data. medRxiv. 2021;doi:10.1101/2020.12.30.20249034.

[12] Graham MS, Sudre CH, May A, Antonelli M, Murray B, Varsavsky T, et al. The effect of SARS-CoV-2 variant B.1.1.7 on symptomatology, re-infection and transmissibility. medRxiv. 2021;doi:10.1101/2021.01.28.21250680.

[13] Lauer SA, Grantz KH, Bi Q, Jones FK, Zheng Q, Meredith HR, et al. The Incubation Period of Coronavirus Disease 2019 (COVID-19) From Publicly Reported Confirmed Cases: Estimation and Application. Annals of Internal Medicine. 2020;172(9):577–582. doi:10.7326/M20-0504.

[14] Park M, Cook AR, Lim JT, Sun Y, Dickens BL. A Systematic Review of COVID-19 Epidemiology Based on Current Evidence. Journal of Clinical Medicine. 2020;9(4). doi:10.3390/jcm9040967.

[15] Ali ST, Wang L, Lau EHY, Xu XK, Du Z, Wu Y, et al. Serial interval of SARS-CoV-2 was shortened over time by nonpharmaceutical interventions. Science. 2020;369(6507):1106–1109. doi:10.1126/science.abc9004.

[16] Jing Q, Liu M, Zhang Z, Fang L, Yuan J, Zhang A, et al. Household secondary attack rate of COVID-19 and associated determinants in Guangzhou, China: a retrospective cohort study. Lancet Infect Dis. 2020;20(10):1141–1150. doi:10.1016/S1473-3099(20)30471-0.

[17] Mistry D, Litvinova M, Pastore y Piontti A, Chinazzi M, Fumanelli L, Gomes MFC, et al. Inferring high-resolution human mixing patterns for disease modeling. Nature Communications. 2021;12(1):323. doi:10.1038/s41467-020-20544-y.

[18] Backer JA, Mollema L, Vos ER, Klinkenberg D, van der Klis FR, de Melker HE, et al. Impact of physical distancing measures against COVID-19 on contacts and mixing patterns: repeated cross-sectional surveys, the Nether-

lands, 201617, April 2020 and June 2020. Eurosurveillance. 2021;26(8). doi:https://doi.org/10.2807/1560-7917.ES.2021.26.8.2000994.
